# Editorial: Magnetic resonance imaging contrast agents: the safety of gadolinium

**DOI:** 10.3389/ftox.2025.1664840

**Published:** 2025-11-07

**Authors:** John Sy, Joshua DeAguero, Brent Wagner

**Affiliations:** 1 Division of Nephrology, Hypertension, and Kidney Transplantation, Department of Medicine, University of California Irvine, School of Medicine, Orange, CA, United States; 2 Kidney Institute of New Mexico, University of New Mexico Health Science Center, Albuquerque, NM, United States; 3 New Mexico VA Healthcare System, Research Service, Albuquerque, NM, United States

**Keywords:** gadolinium (Gd), gadolinium deposition disease, heavy metals, magnetic resonance imaging (MRI), GBCA, GBCA accumulation

Gadolinium-based contrast agents (GBCAs) revolutionized magnetic resonance imaging when introduced in the 1980s, providing clinicians with exceptional clarity in visualizing soft tissue pathology (Lohrke et al., 2016). Their early reputation as benign alternatives to iodinated contrast agents became dogma, bolstered by high diagnostic yield and widespread use in neuroradiology, oncology, and vascular imaging.

Gadolinium (Gd) itself is a rare-earth metal with no known physiologic role, and pharmaceutical formulations bind gadolinium with multidentate organic ligands in an attempt to render it inert and promote elimination. Use of GBCAs increased until the early-mid 2000s when gadolinium was linked to the formation of nephrogenic systemic fibrosis (NSF), a devastating and disfiguring condition manifesting with dermal thickening, joint contractures, and visceral fibrosis in patients with acute or chronic renal dysfunction (Abu-Alfa, 2020). Subsequently, the United States Food and Drug Administration (FDA) and the European Medicines Agency (EMA) mandated boxed warnings on GBCAs, highlighting the risk of systemic fibrosis. The American College of Radiology (ACR) categorized magnetic resonance imaging contrast agents into risk groups for nephrogenic systemic fibrosis (NSF) based on reported cases unconfounded by multiple brand exposures. These categorizations—Group 1 (agents associated with the highest number of NSF cases), Group 2 (fewer or no unconfounded cases), and Group 3 (insufficient data)—largely mirrored market share at the time of classification rather than reflecting intrinsic chemical risk (Starekova et al., 2024). In the aftermath, retrospective assumptions tended to disproportionately implicate agents with larger market shares prior to 2007, inadvertently favoring less widely used agents or those that were still in the approval pipeline—often without direct comparative evidence. Inevitably, given their longer time on the market, our understanding of Group 1 agent toxicity was far more developed than for newer Group 2 agents, for which long-term safety data remain comparatively limited.

Over time, the warnings (and subsequent litigation) drastically reduced the use of GBCAs in patients with high-risk chronic kidney disease and end-stage renal disease leading to a total decrease in the number of cases of systemic fibrosis to the point where researchers may be prone to believing that newer GBCAs are safe and no longer associated with NSF (Maripuri and Johansen, 2020).

**FIGURE 1:**
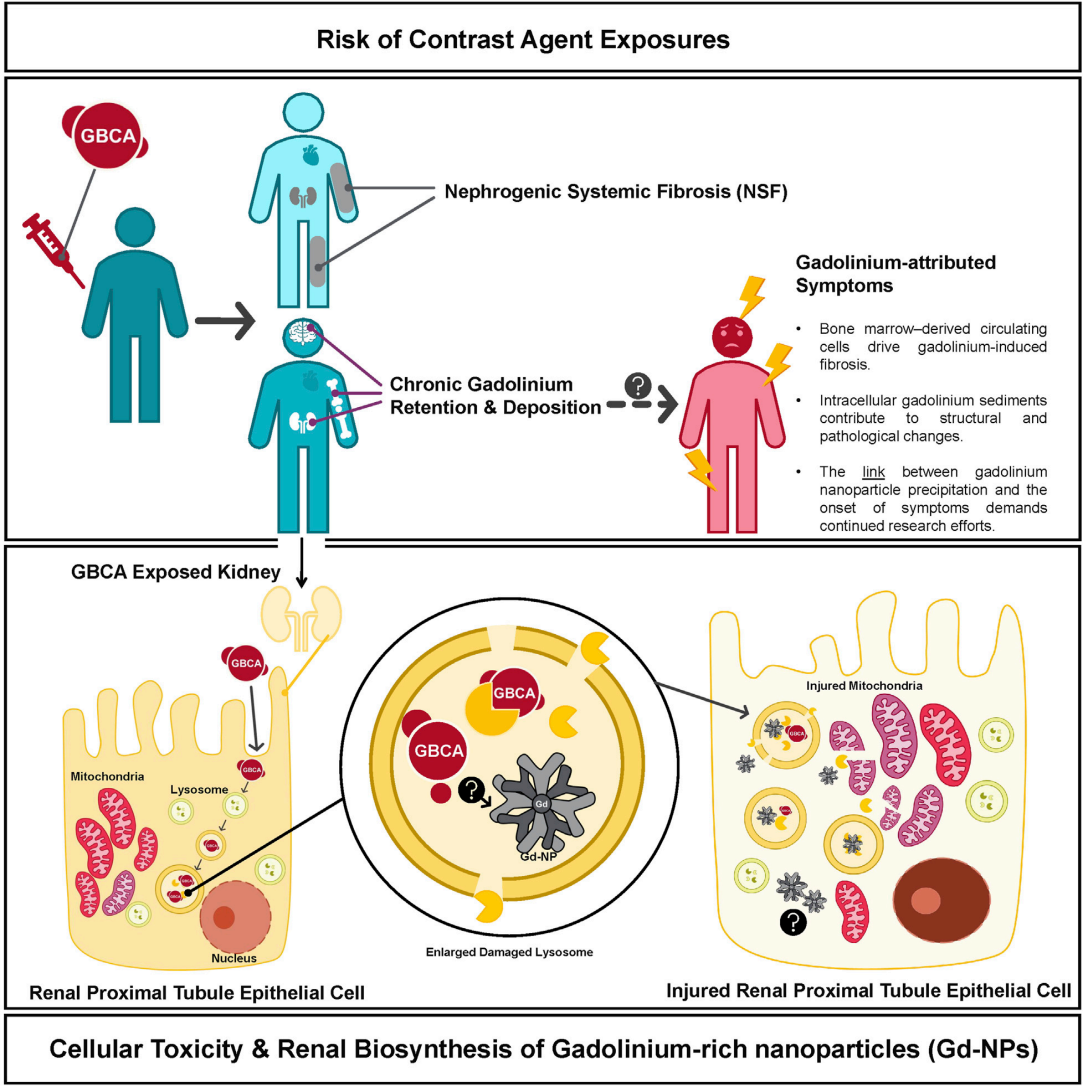
Chronic Gadolinium Deposition and Proposed Mechanisms of Pathophysiology. Gadolinium administration has been linked to the formation of nephrogenic systemic fibrosis and we list proposed mechanisms for the association between Gd contrast exposure and symptoms consistent with Gadolinium Deposition Disease. Intracellular mechanisms of gadolinium toxicity are shown in the lower panel which have also been associated with mitochondrial injury and other cellular damage.

Despite reassuring epidemiological data, *in vivo* studies have increasingly revealed that gadolinium contrast agents may not be biologically inert (Figure 1). In the early to mid-2010s, the effects of chronic gadolinium retention began to be better understood. In a 2016 study, Dr. Tomonori Kanda and colleagues published a study reporting that even in patients with normal renal function, there was an increase in T1 signal intensity in deep brain nuclei that correlated with the cumulative number of GBCA administrations with confirmed gadolinium deposition in both brain and extracranial tissues (Kanda et al., 2016). A couple of years later, Dr. Robert McDonald and his team validated that MRI contrast agent exposure had a legacy of retained gadolinium in central nervous system neurons (including within the nuclei) (Levine et al., 2018). While these studies firmly established the presence of gadolinium in brain tissue, they did not demonstrate a direct link to clinical symptoms. Nonetheless, anecdotal patient reports of persistent pain, cognitive complaints, and other ill-defined symptoms after GBCA exposure have been described in the literature, giving rise to concerns about a condition termed “gadolinium deposition disease” (Ramalho et al., 2016; Layne et al., 2018; Layne et al., 2020). In response to the accumulating evidence of retention and the uncertainty surrounding its clinical implications, the FDA Medical Imaging Drugs Advisory Committee in 2017 recommended expanding warning labels to all GBCAs and required manufacturers to conduct additional human and animal safety studies to better assess potential long-term effects.

Currently, multiple studies have documented gadolinium deposits in bone, brain, and renal tissue, even in individuals with normal renal function and tried to explain the pathologic mechanisms involved (Murata et al., 2016; Bücker et al., 2022; Semelka and Ramalho, 2023; Wagner et al., 2012; Bruno et al., 2021; Do et al., 2019a; Do et al., 2019b; DeAguero et al., 2023; Henderson et al., 2025). Two studies published in this special edition go further to elucidate the mechanism of gadolinium deposition. Investigators at the Kidney Institute of New Mexico identified gadolinium-rich nanoparticles in kidney cells from patients without renal impairment (Cunningham et al.). Notably, they were not dissociated ions but complex aggregates formed after injection. In another study published in this special edition, John Prybylski and his team showed that iron deficiency primes the brain for gadolinium uptake (Prybylski et al.). In rodents, it targets the olfactory bulbs—regions rich in oxidation-reduction activity. Human data echo the rodent models: patients exposed to contrast agents report neuropathic pain, cognitive fog, and crushing fatigue (Semelka and Ramalho, 2023).

While these studies further our understanding of the pathophysiology behind gadolinium deposition disease, they also demand larger scale safety studies. Chelated gadolinium appears inert in solution, but these studies show clinically relevant quantities of gadolinium dissociating from its multidentate ligand cage and lingering, especially in a cellular milieu saturated with agents primed to precipitate rare earth metals. The ACR guidelines appear to exonerate agents that have not been administered at the scale of Magnevist, Omniscan, OptiMARK, and Gadovist, while asserting the safety of Group 2 agents with a level of certainty that may outpace the current evidence. In the absence of a clear mechanistic understanding of gadolinium-related complications, these guidelines rest on assumptions that may be premature.

How does this translate to bedside? Most providers do not understand the mechanisms of gadolinium-induced systemic fibrosis, nor do they know the kinetics of GBCA elimination. Recognizing the limits of our understanding is a fundamental part of scientific inquiry and we encourage clinicians who frequently order MRI studies to not be cavalier with contrast. If a non-contrast study can provide the same diagnostic yield, why should we risk the exposure to a substance with yet potential unforeseen consequences. For clinicians with a deeper understanding, we encourage them to ask these thought-provoking questions: Why does gadolinium retention correlate with symptoms that evade clear nosological categories? Why are we willing to accept the permanence of gadolinium deposits in the brain without demanding mechanistic clarity? These studies here advance our understanding of gadolinium toxicity, though further research is needed to clarify its effects on cellular physiology.

We also encourage policymakers and radiology leadership to reassess the axioms. Group 2 agents are not inert. The burden of proof must lie with those who assert long-term safety, not those who question it.

The patients have spoken and their voice matters. Accounts of long-term gadolinium deposition disease—often derided as anecdotes—signal phenomena our models do not yet explain.

In the end, there are no perfect agents, only trade-offs. But vigilance and transparency should define those trade-offs. We need unbiased registries, longitudinal studies, and mechanistic research to trace the true sequelae of GBCA exposure.
